# Cigarette smoke extract stimulates human pulmonary artery smooth muscle cell proliferation: Role of inflammation and oxidative stress

**DOI:** 10.22038/IJBMS.2022.64170.14133

**Published:** 2022-06

**Authors:** Juan Wang, Le Wang, Xing Chen, Mao-Li Liang, Dong-Hui Wei, Wei Cao, Jing Zhang

**Affiliations:** 1 Department of Respiratory and Critical Care Medicine, Tianjin Medical University General Hospital, Tianjin 300052, China

**Keywords:** Cigarette smoking, Inflammation, NF-_κ_B, Oxidative stress, Pulmonary arterial - hypertension

## Abstract

**Objective(s):**

Cigarette smoke may play a direct role in proliferation of human pulmonary artery smooth muscle cells (HPASMCs). However, the mechanism involved and the effect of interventions remain unclear. We aimed to evaluate the effect of cigarette smoke extract (CSE) on HPASMCs, explore the role of inflammation and oxidative stress, and the effects of Tempol and PDTC in this process.

**Materials and Methods:**

HPASMCs were subjected to normal control (NC), CSE, CSE+Tempol (CSE+T), and CSE+PDTC (CSE+P) groups. Proliferation of HPASMCs was measured by CCK-8 and Western blot. TNF-α, IL-6, MDA, and SOD levels were determined by ELISA and commercial kits. Nuclear translocation of NF-κB p65 was evaluated by western blot.

**Results:**

1%, 2.5%, and 5% CSE all promoted proliferation of HPASMCs, and effect of 1% CSE was the most significant, however, 7.5% and 10% CSE inhibited viability of cells (all *P*<0.05). Compared with the NC group, TNF-α, IL-6, and MDA levels increased, SOD activity decreased (all *P*<0.05), and NF-κB p65 expression in nuclei increased (*P*=0.04) in the CSE group. Tempol and PDTC inhibited the proliferation of HPASMCs induced by CSE (all *P*<0.05). And compared with the CSE group, TNF-α, IL-6, and MDA levels in CSE+T and CSE+P groups decreased, while SOD activity increased (all *P*<0.05). Tempol reduced the expression of NF-κB p65 in nuclei but did not achieve a significant difference (*P*=0.08). PDTC inhibited the nuclear translocation of NF-κB p65 (*P*=0.03).

**Conclusion:**

CSE stimulates HPASMCs proliferation in a certain concentration range. The CSE-induced proliferation of HPASMCs involved excessive inflammatory response and oxidative stress. Tempol and PDTC attenuate these effects of CSE on HPASMCs.

## Introduction

Chronic obstructive pulmonary disease (COPD) is a kind of chronic airway and lung disease with respiratory symptoms such as persistent cough and expectoration. It has been generally accepted and acknowledged that COPD with significant characteristic of irreversible airflow limitation has far-reaching effects on cardiac function and gas exchange, which could initiate various comorbid diseases (1). Pulmonary hypertension (PH) is one of the most common complications characterized by the rise in pulmonary vascular resistance and pulmonary arterial pressure, eventually resulting in right ventricular hypertrophy, functional decline, failure, and even death (2). The prevalence of COPD complicated with PH varies from 20%–91% (3, 4), and increases with the severity of COPD (5). It is universally considered that COPD complicated with PH results from hypoxia in the process of illness. Chronic hypoxia stimulates pulmonary vasoconstriction and causes remodeling of pulmonary small vessels. Interestingly, some studies have found that pulmonary tissues of COPD had obvious pathological reconstruction in the early days even before hypoxia appeared (6-8). Moreover, it has been declared that cigarette smoke may be directly involved in the proliferation of vascular smooth muscle cells in the bovine thoracic aorta, human greater saphenous vein, and aortic and iliac arteries (9). It was also reported that cigarette smoke could directly induce production of vasoactive mediators controlling dynamic vasoconstriction and vasodilation (8). All these findings showed cigarette smoke may directly cause remodeling of pulmonary vessels and PH. Nevertheless, the underlying mechanism and effect of interventions are still poorly understood. 

Pulmonary vascular remodeling is a landmark pathophysiological basis for the development of PH, in which the increased proliferation of pulmonary artery smooth muscle cells (PASMCs) is a pivotal contributor. Abnormal proliferation of PASMCs is believed to be responsible for medial hypertrophy, artery remodeling, and vascular lumen narrowing, which is the typical pathological manifestation of PH (10). There is evidence in the literature that inflammation and oxidative stress were considered to play key intermediary roles in the development of hypoxia-induced PH (11, 12). However, to the best of our knowledge, whether cigarette smoke extraction (CSE) induces excessive inflammation and oxidative stress, and its roles in the proliferation of human pulmonary artery smooth muscle cells (HPASMCs) have not as yet been elucidated clearly. 

Herein, we aimed to clarify the effect of CSE on HPASMCs, investigate whether excessive inflammation and oxidative stress are involved in this pathological process, and explore the effect of interventions, such as 4-hydroxy-2,2,6,6-tetramethylpiperidine (Tempol) and pyrrolidine dithiocarbamate (PDTC) in this process.

## Materials and Methods

***Preparation of CSE solution***

Commercial filtered cigarettes (Zuanshi, Zhangjiakou Cigarette Factory, China), which contain 11 mg tar, 0.9 mg nicotine, and 12 mg carbon monoxide, were used. CSE solution was prepared as described by Oltmanns *et al*. with a few modifications (13). Briefly, smoke derived from one cigarette was slowly dissolved in 10 ml of preheated phosphate buffer solution (PBS, pH=7.4) under suction and driving of 50 ml syringes at room temperature. The resulting solution, which was considered pure stoste, was adjusted to pH 7.4 with NaOH and filtered through a 0.22 μm pore filter before diluting it into solutions of different concentrations with Dulbecco’s modified Eagle’s medium (DMEM, Gibco Life Technologies Inc, Rockville, MD, USA). Finally, the CSE solutions of different concentrations ranging from 0% to 10% (0%, 1%, 2.5%, 5%, 7.5%, and 10%) were used to evaluate the effect of CSE on HPASMCs. 

***Cell culture and treatment***

HPASMCs (Otwo Biotech, Shenzhen, China) were cultured in DMEM supplemented with 10% fetal bovine serum (FBS), 100 U/ml penicillin, and 100 ug/ml streptomycin in a 5% CO_2_ and 37 °C incubator with saturated humidity. The medium was replaced every other day. HPASMCs were treated with varying concentrations of CSE solutions for 24 hr, with or without the anti-oxidant, Tempol (0.4 mmol/L, Sigma-Aldrich), or the NF-κB inflammatory channel blocker, PDTC(50 umol/L, Abcam).

***Cell proliferation analysis***

The effects of CSE on viability and proliferation of HPASMCs waere examined by Cell Counting Kit-8 (CCK-8) assay according to the kit instructions (Nanjing Jiancheng Bioengineering Research Institute, China). Cells (1*10^4^/well) were seeded in 96-well plates in replicates of three. After 24 hr of different treatments, the absorbance at 490 nm was recorded.

***Western blot analysis***

After being cultured for 24 hr, HPASMCs were lysed with radioimmunoprecipitation (RIPA) buffer with 1 mM phenylmethylsulfonyl fluoride (PMSF) on ice for 30 min. The cell lysates were sonicated and then centrifuged at 12,000 rpm for 15 min at 4 °C, and the insoluble fraction was discarded. Cytoplasmic and nuclear protein was extracted according to the protocol of the Nuclear and Cytoplasmic Protein Extraction Kit (Sangon Biotech, Shanghai, China). The protein concentration was measured using the BCA Protein Assay Kit (Thermo, USA). The samples were separated on 10% sodium dodecyl sulfate-polyacrylamide gels and transferred to polyvinylidene difluoride membranes. Then the membranes were blocked with 5% non-fat milk for 1 hr at room temperature, followed by overnight incubation at 4 °C with primary antibodies against the following proteins: H_3_ antibody (1:1000), β-actin antibody (1:1000), α-SMA antibody (1:1000), NF-κB antibody (1:1000). After overnight incubation, the membranes were incubated with appropriate HRP-labeled secondary antibodies at a dilution of 1:3000. The ECL detection system (Thermo, USA) was used to detect the signals on the membranes.

***Enzyme-linked immunosorbent assay***

The levels of tumor necrosis factor-α (TNF-α) and interleukin-6 (IL-6) were determined in the extracted supernatant with the use of the double-antibody sandwich enzyme-linked immunosorbent assay (ELISA, R&D System, Los Angeles, USA) according to the protocol.

***Assay of MDA and SOD***

The contents of malondialdehyde (MDA) and superoxide dismutase (SOD) were measured using commercial kits (Nanjing Jiancheng BioengineeringResearch Institute, China) and analyzed with a spectrophotometer according to the manufacturer’s instructions.

***Statistical analysis***

SPSS 25.0 software package (SPSS Inc., Chicago, IL, USA) was used for statistical analysis and illustration. All results in the study were expressed as mean±standard deviation. One-way analysis of variance was performed for whole difference among groups, and the difference between two groups was compared using the Student t-test. Two-sided *P*<0.05 was considered statistically significant.

## Results

***Effect of different concentrations of CSE on HPASMCs proliferation***

**Figure 1:**
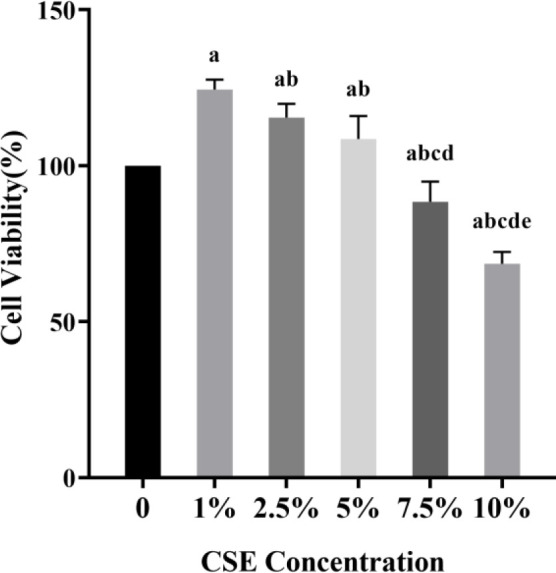
The HPASMCs were treated with increasing concentrations of CSE for 24 hr. Cell proliferation was analyzed by CCK-8 assay. The data were shown as mean±standard deviation from five duplicated experiments. ^a^*P*<0.05 compared with NC group,^ b^*P*<0.01 compared with 1% CSE group, ^c^*P*<0.01 compared with 2.5% CSE group,^ d^*P*<0.01 compared with 5% CSE group, ^e^*P*<0.01 compared with 7.5% CSE group

HPASMCs: human pulmonary artery smooth muscle cells; NC: normal control; CSE: cigarette smoke extract; CCK-8: Cell Counting Kit-8

To investigate the effect of different concentrations of CSE on cell proliferation, HPASMCs were stimulated with 0%, 1%, 2.5%, 5%, 7.5%, and 10% CSE solutions for 24 hr, respectively. As shown in Figure 1, the 1%, 2.5%, and 5% CSE treatments all caused a significant increase in cell growth compared with the NC group, and the effect of 1% CSE was the most significant (all *P*<0.05). However, 7.5% and 10% CSE significantly inhibited cell viability (all *P*<0.05). Therefore, 1% CSE was used for further experiments.

***Effect of Tempol and PDTC on HPASMCs proliferation promoted by 1% CSE***

**Figure 2:**
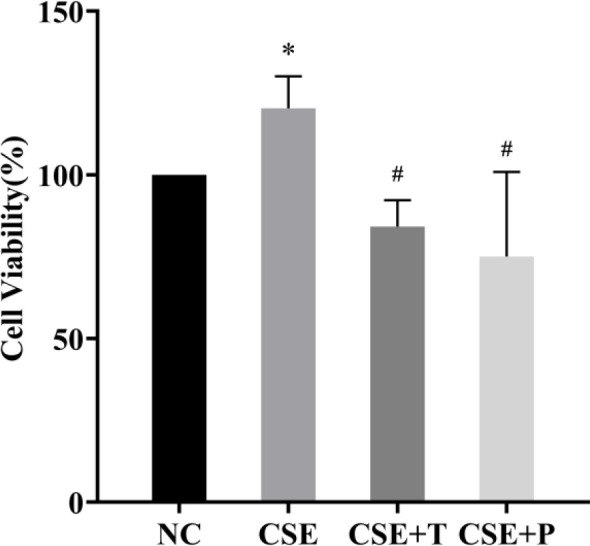
HPASMCs were pretreated with Tempol or PDTC before exposure to 1% CSE. Cell viability was analyzed by CCK-8 assay. The data were shown as mean±standard deviation from three replicated experiments. *P<0.05 compared with NC group, #*P*<0.05 compared with 1% CSE group

PDTC: pyrrolidine dithiocarbamate; HPASMCs: human pulmonary artery smooth muscle cells; NC: normal control; CSE: cigarette smoke extract; CCK-8: Cell Counting Kit-8

**Figure 3:**
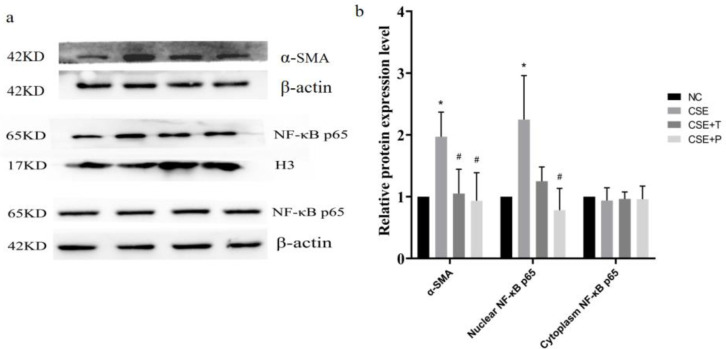
CSE induced up-expression of α-SMA and nuclear translocation of NF-κB p65 in HPASMCs. a: Western blot analysis showed the expression level of α-SMA, Nuclear NF-κB p65, Cytoplasm NF-κB p65, β-actin, and H3. b: Summarized data showed the average protein level of α-SMA and NF-κB p65 in the nuclear and cytoplasm fraction. *P<0.05 compared with NC group, #*P*<0.05 compared with 1% CSE group

Within the 24-hr 1% CSE exposure, cell viability was significantly increased in the CSE group compared with that in the NC group (*P*=0.02). Tempol and PDTC treatment significantly inhibited the increase of cell growth induced by CSE (all *P*<0.05, Figure 2). Consistent with this, a significant up-regulation of α-SMA, a proliferation-related protein, in the CSE group was observed by Western blotting, compared with that in the NC group (*P=*0.01, Figure 3). However, Tempol and PDTC treatment inhibited the increased expression of α-SMA in HPASMCs induced by CSE (all *P*<0.05, Figure 3).

***Comparisons of inflammatory factors in the supernatant***

**Figure 4:**
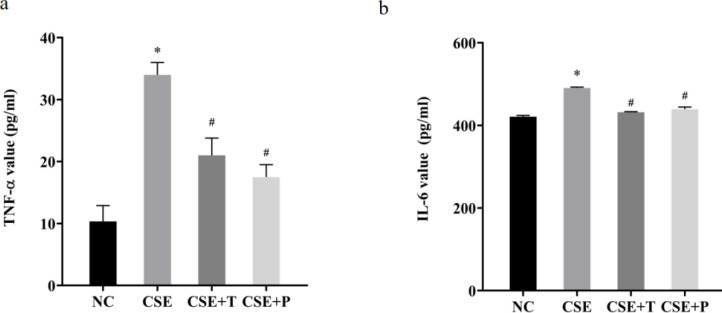
Effect of CSE on TNF-α (a) and IL-6 (b) levels in the supernatants of HPASMCs with or without Tempol and PDTC. The data were expressed as the mean±standard deviation of three replicated experiments.*P<0.01 compared with NC group, #*P*<0.01 compared with 1% CSE groupC: normal control; PDTC: pyrrolidine dithiocarbamate; HPASMCs: human pulmonary artery smooth muscle cells; CSE: cigarette smoke extract

Within the 24-hr 1%CSE exposure, TNF-α and IL-6 levels in the CSE group both significantly increased compared with that in the NC group (all *P*<0.01, Figure 4). The anti-oxidant, Tempol, and the NF-κB inflammatory channel blocker, PDTC, both significantly reduced the levels of TNF-α and IL-6 induced by CSE exposure (all *P*<0.01, Figure 4).

***Comparisons of oxidative stress markers in the supernatant***

**Figure 5:**
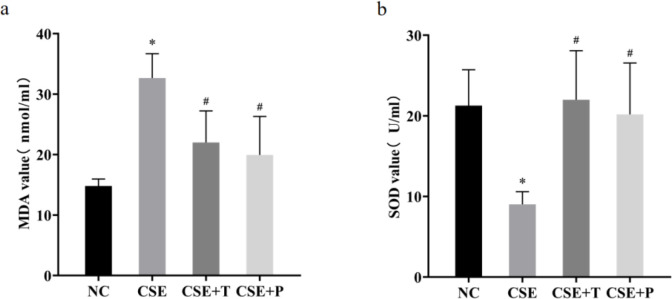
Levels of MDA concentration (a) and SOD activity (b) in different exposure conditions. The data were expressed as the mean±standard deviation of three replicated experiments.*P<0.05 compared with NC group, #*P*<0.05 compared with 1% CSE group

MDA: malondialdehyde; SOD: superoxide dismutase; NC: normal control; CSE: cigarette smoke extract

To determine the oxidative stress response of HPASMCs to CSE exposure, the levels of SOD activity and MDA concentration were analyzed in the supernatant. The results showed that CSE decreased the levels of SOD activity, and increased the levels of MDA concentration (all *P*<0.05, Figure 5). Tempol and PDTC significantly reduced this CSE-induced damage (all *P*<0.05, Figure 5).

***Comparison of NF-*** *ĸ* ***B expression levels in HPASMCs***

CSE induced nuclear translocation of NF-κB. As shown in Figure 3, exposure of HPASMCs to 1% CSE had little effect on the expression level of NF-κB p65 in the cytoplasm (*P*=0.97), but significantly increased the expression level of NF-κB p65 in nuclei (*P*=0.04). Tempol decreased the expression level of NF-κB p65 in nuclei compared with that in the CSE group but failed to achieve a statistically significant difference (*P*=0.08). PDTC markedly inhibited the nuclear translocation of NF-κBp65 (*P*=0.03).

The data were shown as mean±standard deviation from three repeated experiments

HPASMCs: human pulmonary artery smooth muscle cells; NC: normal control; CSE, cigarette smoke extract

## Discussion

Cigarette smoking is a major public health problem, responsible for killing more than seven million people every year globally (14), associated with various kinds of diseases, including vascular diseases, such as atherosclerosis and PH (15). Cigarette smoke is a complex chemical mixture that contains over 4000 different compounds, in which 158 of the chemical constituents are identified to be toxic hazards, and 15 individual chemical constituents contribute to cardiovascular dysfunction (16). Studies in both animal models and human patients have shown that cigarette smoke exerted a direct effect on pulmonary vascular structure, resulting in pulmonary vascular remodeling and PH (17, 18). Proliferation of vascular smooth muscle cells is considered to play a key role in the occurrence and development of vascular diseases. Abnormal vascular smooth muscle cell proliferation leads to vascular remodeling, medial hypertrophy of the vessel wall, and vascular lumen narrowing, all of which contribute to the development of PH (19, 20). In this study, we have shown that lower concentrations of CSE (1%, 2.5%, and 5%) stimulated the proliferation of HPASMCs, whereas higher concentrations of CSE (7.5% and 10%) were inhibitory as a result of cytotoxicity. These results are consistent with previous findings, although the concentration of CSE with the best proliferative effect on cells is different (21, 22). This may be related to different CSE preparation methods, cell types, and cell culture conditions (21).

A substantial body of evidence has affirmed that PH was linked to inflammation (11, 23-26). The inflammatory factors included IL-1**β**, IL-2, IL-4, IL-5, IL-6, IL-8, IL-10, IL-12p70, IL-13, TNF-**α**, etc (11,27). The studies on human patients showed the serum levels of IL-6 in PH patients were significantly higher than that in normal controls(28); and the levels were also related to the survival of patients(25, 27,29). Moreover, the plasma levels of IL-6 were higher in COPD patients complicated with PH compared with pure COPD patients, and the levels correlated with mean pulmonary arterial pressure (30). Bhargava *et al*. declared that the expression of IL-6 mRNA and bioactivity of IL-6 were increased in the lung of monocrotaline-induced PH and right ventricular hypertrophy (RVH) rats. When the rats were treated with dexamethasone, the IL-6 levels decreased, and the pulmonary pressures and RVH attenuated compared with pre-treatment (31). Steiner *et al*. found that there was enhanced muscularization of distal arterioles and occlusive neointimal angioproliferative lesions in rats over-expressing IL-6, which were consistent with the early pathological manifestations of PH and RVH (32). Similarly, elevated serum levels of TNF-α were described in PH patients, and the levels were also associated with quality of life-related symptoms (25, 27). In addition, COPD patients with PH showed significantly higher TNF-α levels than COPD patients without PH (33). Several studies have shown that over-expression of TNF-α could result in chronic pulmonary inflammation and PH (34, 35). However, TNF-**α** antagonists showed an ameliorated effect on pulmonary hemodynamic, RVH, and pulmonary inflammation in rats with monocrotaline-induced PH and in pigs with endotoxemic-shock-induced PH (36-38). Smoking is associated with the release and inhibition of pro-inflammatory and anti-inflammatory mediators. Cigarette smoke has been shown to increase the production of a variety of pro-inflammatory cytokines, such as TNF- α and IL-6, and reduce the levels of anti-inflammatory cytokines, such as IL-10 (39). NF-κB is a pro-inflammatory transcription factor controlling many genes which are important for immunity, inflammation, cell proliferation, and apoptosis. Under physiological conditions, NF-κB dimer was sequestered in the cytoplasm in an inactive form bounding to its inhibitory protein, IκB. Under pathological conditions, IκB kinase was activated, then IκB was phosphorylated, and the released NF-κB dimer translocated to the nucleus, in which it induced transcription of NF-κB target genes (40). It has been reported that NF-κB played key roles in pathological processes of PH, including monocrotaline-induced PH, growth factors (PDGF, bFGF, EGF, and IGF-1)-induced PH, and chronic hypoxia-induced PH (41-44). In accordance with this, our data showed that CSE exposure caused nuclear translocation of NF-κB in the process of HPASMC proliferation. Meanwhile, our results showed that IL-6 and TNF-α levels were increased in the CSE exposure group versus the NC group, suggesting that inflammation may be involved in vascular smooth muscle cell proliferation under CSE stimulation through NF-κB pathway activation.

Another important factor involved with PH was oxidative stress. Increased oxidative stress was considered to play a pivotal role in the development of hypoxia-induced PH (45, 46). Reactive oxygen species (ROS) was the most important effector in excessive oxidative damage in the pathogenesis of PH, which regulates the release of several vasoactive factors, such as ET-1, TXA-2, and prostacyclin, which can influence vasomotor and lead to vascular remodeling (47-49). ROS has been proven to increase in both *in vitro* and *in vivo* models of chronic hypoxia-induced vascular smooth muscle cell proliferation and pulmonary vessel remodeling, and inhibition of ROS production has been shown to attenuate PH (50-52). Oxidative stress can be reflected by several markers. The lipid peroxidation product, MDA, can indirectly reflect cellular oxidative stress levels, while SOD can protect the cells against the potential damage from superoxide radicals via catalyzing the conversion of superoxide radicals to hydrogen peroxide. COPD patients had higher serum MDA concentrations compared with healthy subjects (53). Moreover, the serum MDA levels in COPD patients with PH were elevated compared with that in COPD patients without PH, while the SOD levels were reduced in COPD patients with PH compared with that in pure COPD patients. Additionally, the serum MDA levels were positively correlated with 3-year PH incidence, and negatively correlated with 3-year survival rate in COPD patients; however, serum SOD levels were negatively correlated with 3-year PH incidence, and positively correlated with 3-year survival rate in COPD patients(54). Previous studies have demonstrated that billions of free radicals and chemicals in cigarettes could generate ROS through the redox cycle, causing excessive oxidative stress in the lungs and imposing an undue oxidative burden (55). It has been reported that ROS can cause oxidative damage to the cell’s lipids, proteins, and DNA, and may be involved in the development of PH (56). Our results showed that the CSE group had higher levels of MDA concentrations and lower levels of SOD activity as compared with those in the NC group, indicating that excessive oxidative stress would be present in vascular smooth muscle cell proliferation upon CSE stimulation.

Our current results showed that the levels of inflammatory factors, oxidative stress markers, and proliferation-associated indicators were all improved in the CSE+T/P group as compared with that in the CSE group. Tempol is a superoxide scavenger, which can reduce the damage of exogenous ROS to cultured cells (57). It has been reported that Tempol reduced superoxide production and improved vascular endothelial function (58, 59). Tempol can also decrease pulmonary arterial hypertension in hypoxia-induced PH rats (12). Here, our data showed that Tempol was effective in alleviating the proliferation of HPASMCs under CSE stimulation by diminishing oxidative stress and inflammatory response. Meanwhile, we also declared that PDTC, which inhibits the activation and translocation of NF-κB by repressing the phosphorylation and degradation of IκB (60), resulted in markedly reduced expression/production of TNF-α, IL-6, and MDA, and improved production of SOD, which contributed to decreasing the proliferating effect of CSE on HPASMCs. 

## Conclusion

In summary, CSE in a certain concentration range could stimulate the proliferation of HPASMCs, which is one of the pathogenesis of PH, through aggravating inflammatory responses and oxidative stress. Application of Tempol and PDTC could markedly reduce the CSE-induced proliferation of HPASMCs by attenuating the above process.

## Authors’ Contributions

The work presented in this article was carried out through collaboration between all authors. JW and LW Made the initial hypothesis. All authors participated in defining the research theme and providing the proposal. JW, LW, XC, MLL, and DHW Performed the experiments. JW, LW, and XC Interpreted the data and wrote and edited the article. JC and JZ Supervised the work. All authors edited and approved the article.

## Conflicts of Interest

There are no conflicts of interest to declare.
